# Pathological Progression Induced by the Frontotemporal Dementia-Associated R406W Tau Mutation in Patient-Derived iPSCs

**DOI:** 10.1016/j.stemcr.2019.08.011

**Published:** 2019-09-19

**Authors:** Mari Nakamura, Seiji Shiozawa, Daisuke Tsuboi, Mutsuki Amano, Hirotaka Watanabe, Sumihiro Maeda, Taeko Kimura, Sho Yoshimatsu, Fumihiko Kisa, Celeste M. Karch, Tomohiro Miyasaka, Akihiko Takashima, Naruhiko Sahara, Shin-ichi Hisanaga, Takeshi Ikeuchi, Kozo Kaibuchi, Hideyuki Okano

**Affiliations:** 1 Department of Physiology, School of Medicine, Keio University, 35 Shinanomachi, Shinjuku-ku, Tokyo 160-8582, Japan; 2 Department of Biomedical Chemistry, School of International Health, Graduate School of Medicine, University of Tokyo, 7-3-1 Hongo, Bunkyo-ku, Tokyo 113-8654, Japan; 3 Department of Cell Pharmacology, Graduate School of Medicine, Nagoya University, 65 Tsurumai, Showa, Nagoya, Aichi 466-8550, Japan; 4 Department of Functional Brain Imaging Research, National Institute of Radiological Sciences, 4-9-1 Anagawa, Inage, Chiba 266-8555, Japan; 5 Department of Psychiatry and Hope Center for Neurological Disorders, Washington University in St. Louis, St. Louis, MO 63110, USA; 6 Department of Neuropathology, Faculty of Life and Medical Sciences, Doshisha University, Kyotanabe-shi, Kyoto 610-0394, Japan; 7 Faculty of Science, Gakushuin University, Toshima-ku, Tokyo 171-8588, Japan; 8 Department of Biological Sciences, Graduate School of Science, Tokyo Metropolitan University, 1-1 Minami-Osawa, Hachioji-shi, Tokyo 192-0397, Japan; 9 Department of Molecular Genetics, Brain Research Institute, Niigata University, 1-757 Asahimachidori, Chuo-ku, Niigata 951-8585, Japan

**Keywords:** iPSC, tau, neurodegenerative disease, disease modeling, FTD

## Abstract

Mutations in the microtubule-associated protein tau (*MAPT*) gene are known to cause familial frontotemporal dementia (FTD). The R406W tau mutation is a unique missense mutation whose patients have been reported to exhibit Alzheimer’s disease (AD)-like phenotypes rather than the more typical FTD phenotypes. In this study, we established patient-derived induced pluripotent stem cell (iPSC) models to investigate the disease pathology induced by the R406W mutation. We generated iPSCs from patients and established isogenic lines using CRISPR/Cas9. The iPSCs were induced into cerebral organoids, which were dissociated into cortical neurons with high purity. In this neuronal culture, the mutant tau protein exhibited reduced phosphorylation levels and was increasingly fragmented by calpain. Furthermore, the mutant tau protein was mislocalized and the axons of the patient-derived neurons displayed morphological and functional abnormalities, which were rescued by microtubule stabilization. The findings of our study provide mechanistic insight into tau pathology and a potential for therapeutic intervention.

## Highlights

• Phenotype analysis of FTD R406W mutant tau using iPSC-derived neurons
• Homogeneous neuronal population obtained via dissociation of cerebral organoids
• Altered phosphorylation and fragmentation state of R406W mutant tau
• Multiple axonal defects in patient neurons rescued with microtubule stabilization

## Graphical Abstract

**Figure:**
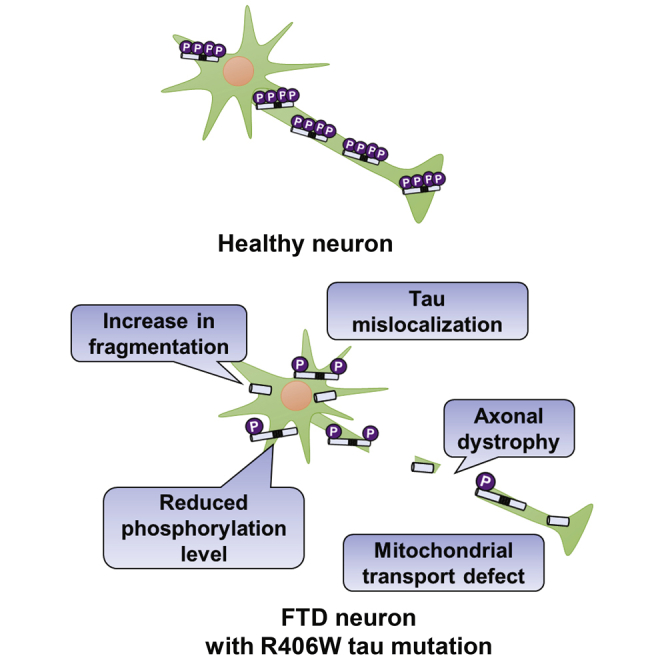

## Teaser

In this article, Nakamura and colleagues establish an iPSC-derived neuronal model from frontotemporal dementia patients with the tau R406W mutation and gain insight into the disease pathology. Here, the R406W mutant tau exhibited reduced phosphorylation levels and was prone to fragmentation by calpain. Furthermore, the patients’ neurons displayed multiple axonal defects caused by microtubule destabilization.

## Introduction

Frontotemporal dementia (FTD) is one of the most common types of early-onset dementia after Alzheimer's disease (AD). In some familial cases, it is caused by mutations in the microtubule-associated protein tau (*MAPT*) gene, which encodes the tau protein (Hutton et al., 1998, Poorkaj et al., 1998, Spillantini et al., 1998). Over 50 mutations on the *MAPT* gene have been reported to cause FTD (Ghetti et al., 2015), and the patients exhibit diverse clinical phenotypes (Ghetti et al., 2011). The R406W missense mutation is one such pathological mutation located on exon 13 of the *MAPT* gene (Hutton et al., 1998, Rizzu et al., 1999, Van Swieten et al., 1999). Interestingly, patients with this mutation have been reported to exhibit AD-like phenotypes: early memory impairment is a primary presenting feature, while the more typical FTD symptoms, including changes in social behavior and personality, as well as motor symptoms (Foster et al., 1997), are less predominant or not seen at all (Ikeuchi et al., 2011).

As of today, there is no effective treatment for FTD patients. It is crucial to gain a better understanding of the disease pathogenesis for the development of novel therapeutic strategies. Previous studies using transgenic murine models and postmortem patient brains have yielded significant insights into the disease mechanism, but each have their own shortcomings. The former model has not been able to completely recapitulate human disease pathology, possibly due to species differences and overexpression of the tau transgene in irrelevant brain areas (Denk and Wade-Martins, 2009), and the latter cannot model disease onset or progression, and cannot be easily accessed for research purposes due to ethical limitations. Thus, there is a need for a model that is readily accessible and can accurately recapitulate the disease pathogenesis. Recently, considerable attention has been given to the potential of human induced pluripotent stem cell (iPSC) technology (Takahashi et al., 2007) for disease modeling. Human iPSCs have the potential to differentiate into neurons, which have previously been difficult to obtain. Using this model could thereby provide us with a deeper understanding of the molecular mechanism of the disease onset and progression.

Previously there have been reports on the generation of *MAPT* R406W iPSC lines, but those conducting phenotype analysis at the molecular level have been limited (Jiang et al., 2018, Nimsanor et al., 2016a, Nimsanor et al., 2016b, Rasmussen et al., 2016a, Rasmussen et al., 2016b). On the one hand, Imamura et al. (2016) has observed an accumulation of misfolded tau, calcium dysregulation, and neuronal cell death in FTD iPSC models, including those carrying the R406W mutation, but as these phenotypes were commonly found in neurons with other tau mutations as well, the pathological mechanism specific for the R406W mutation remains unexplored.

Here, we sought to establish a model that can recapture the disease pathogenesis induced by the R406W mutation, as a basis for therapeutic development for tauopathies including FTD and AD. Using neurons differentiated from patient-derived iPSCs and their corresponding isogenic lines, we identified the biochemical changes of tau induced by the R406W mutation and their effects at the cellular level.

## Results

### Generation and Characterization of the *MAPT* R406W iPSCs

**Figure 1:**
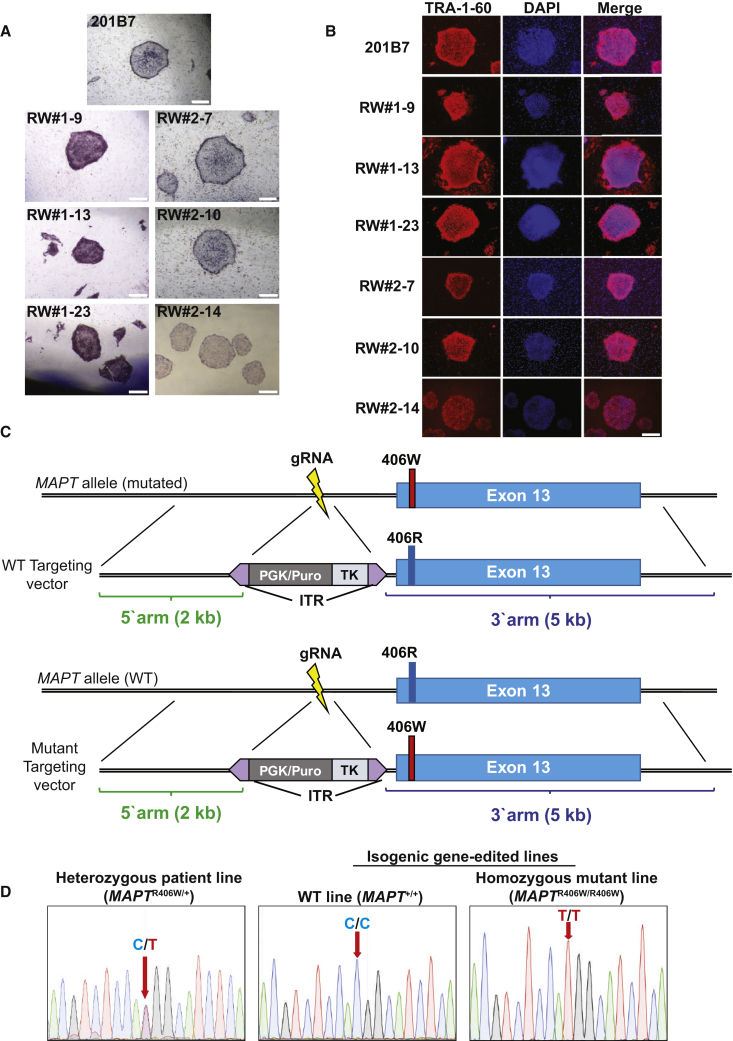
Generation and Characterization of iPSCs from FTD *MAPT* R406W Patients (A and B) Immunofluorescence for pluripotency markers alkaline phosphatase (A) and TRA-1-60 (B). Representative clones from each patient are shown. Scale bars, 500 μm. (C) Schematic diagram of the construction of targeting vectors. Top: construct of the WT targeting vector for the generation of WT lines. Bottom: construct of the mutant targeting vector for the generation of homozygous mutant lines. (D) DNA sequence of the mutation site in the heterozygous patient line (*MAPT*^R406W/+^) and in the gene-edited isogenic lines (WT line: *MAPT*^+/+^, homozygous mutant line: *MAPT*^R406W/R406W^). See also Figure S1.

*MAPT* R406W iPSCs were established from two Japanese FTD patients (patients #1 and #2) of the same pedigree, whose primary symptom was memory impairment (Ikeuchi et al., 2011; Table S1). DNA sequencing confirmed that the patients were heterozygous for the mutation (*MAPT*^R406W/+^, Figure 1D; Ikeuchi et al., 2011). The patients had no other mutations besides the *MAPT* R406W mutation, as previously confirmed (Ikeuchi et al., 2011).

The iPSCs were generated by using the integration-free episomal vector system, as described previously (Ichiyanagi et al., 2016, Nakamoto et al., 2018, Okita et al., 2013). PCR analysis confirmed the removal of the episomal vectors from several clones (Figure S1A). These clones stained positively for the pluripotency markers alkaline phosphatase (Figure 1A) and TRA-1-60 (Figure 1B) and were karyotypically normal (Figure S1B).

We utilized another *MAPT* R406W iPSC line generated with Sendai virus vectors from a symptomatic subject of an unrelated pedigree (patient #3), who was also heterozygous for the mutation (Jiang et al., 2018; Table S1). The 201B7 iPSC line, derived from a healthy subject, was obtained for use as a control for subsequent experiments (Takahashi et al., 2007).

### Generation of Isogenic iPSC Lines with CRISPR/Cas9

Next, we utilized CRISPR/Cas9 technology to manipulate the mutation site for the generation of isogenic lines (Cong et al., 2013) in order to reduce the variability of experiments caused by genetic heterogeneity. Targeting vectors were designed to include the mutation site on the 3′ arm and a selection cassette in between the two arms, with PiggyBac inverted terminal repeats at both ends to enable footprint-free excision (Figure 1C). Transfection experiments were performed, and colonies that survived drug selection were picked up and analyzed. Consequently, DNA sequencing revealed the successful manipulation of the mutation site, confirming the generation of isogenic wild-type (WT) *MAPT*^+/+^ lines and homozygous mutant (*MAPT*^R406W/R406W^) lines (Figure 1D). Finally, the gene-edited clones were transfected with the PiggyBac transposase for the excision of the selection cassette (Nakamoto et al., 2018). DNA-sequencing analysis in the manipulated region confirmed that these isogenic clones were free of genomic indels and other unintended mutations (Figure S1C).

### Establishment of iPSC-Derived Neuron-Rich Culture via Dissociation of Cerebral Organoids

**Figure 2:**
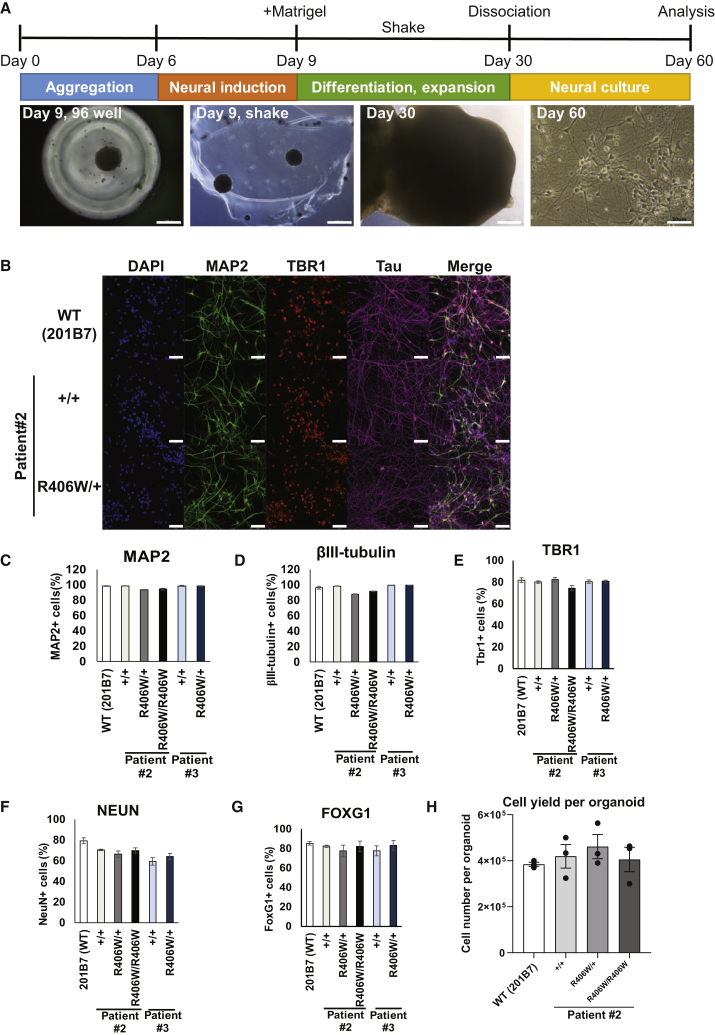
Neural Differentiation of R406W iPSCs via Cerebral Organoid Dissociation (A) Schematic timeline for neuronal differentiation of iPSCs using the protocol for cerebral organoid induction. Organoids were dissociated into cortical neurons and cultured on typical two-dimensional plates. All analyses were performed after 60 days of culture. Scale bar, 500 μm (day 9, day 30), 50 μm (day 60). (B) Immunofluorescence of iPSC-derived cortical neurons using neuronal markers MAP2 and βIII-tubulin, forebrain marker TBR1, tau, and DAPI for nuclear staining. Scale bars, 50 μm. (C–G) Quantification of the percentage of cells expressing pan-neuronal markers MAP2 (C), βIII-tubulin (D), and NeuN (G), and forebrain markers TBR1 (E) and FOXG1 (F) in each iPSC-derived neuronal line (n = 3 independent experiments). (H) Quantification of the number of live cells obtained from one dissociated organoid (n = 3 independent experiments). Error bars indicate mean ± SEM.

The iPSC lines were then subjected to neuronal differentiation for subsequent analysis (Figure 2A). We utilized the method for inducing cerebral organoids (Kadoshima et al., 2013, Lancaster et al., 2013), since previous reports have implicated that three-dimensional (3D) cultures may accelerate disease pathology compared with conventional two-dimensional (2D) cultures (Choi et al., 2014). However, because cerebral organoids consist of a heterogeneous population of cells (Quadrato et al., 2017) and the cells within the organoids are densely packed, we found that the use of cerebral organoids as a whole was not suitable for biochemical analyses and morphological observations of neurons. Thus, we devised a method to isolate a pure population of cortical neurons from the organoids. After 30 days of culture, we dissociated the organoids onto typical 2D culture dishes and cultured them for an additional 30 days, at which point these cultures were evaluated by immunofluorescence. Lines that were successfully induced into organoids and dissociated consisted of neurons positive for MAP2, TBR1, and tau (Figure 2B). Quantification revealed that all the lines evaluated were >85% positive for neuronal markers MAP2 and βIII-tubulin (Figures 2C and 2D), and also highly positive for forebrain (cortical neuron) markers TBR1 and FOXG1, as well as NeuN, indicating the presence of mature neurons (Figures 2E–2G), with similar differentiation efficiency among lines. The yield of live cells from one dissociated organoid of any given line was approximately 4 × 10^5^ cells (equivalent to the number of cells plated onto one well of a 12-well plate), the majority of which we assumed to be neurons (Figure 2H).

Unfortunately, because all the iPSC lines derived from patient #1 did not efficiently differentiate into neurons with our protocol, we focused on using iPSC-derived neurons from patients #2 and #3 for the subsequent analyses.

### R406W Mutant Tau Is Less Phosphorylated by Multiple Kinases

**Figure 3:**
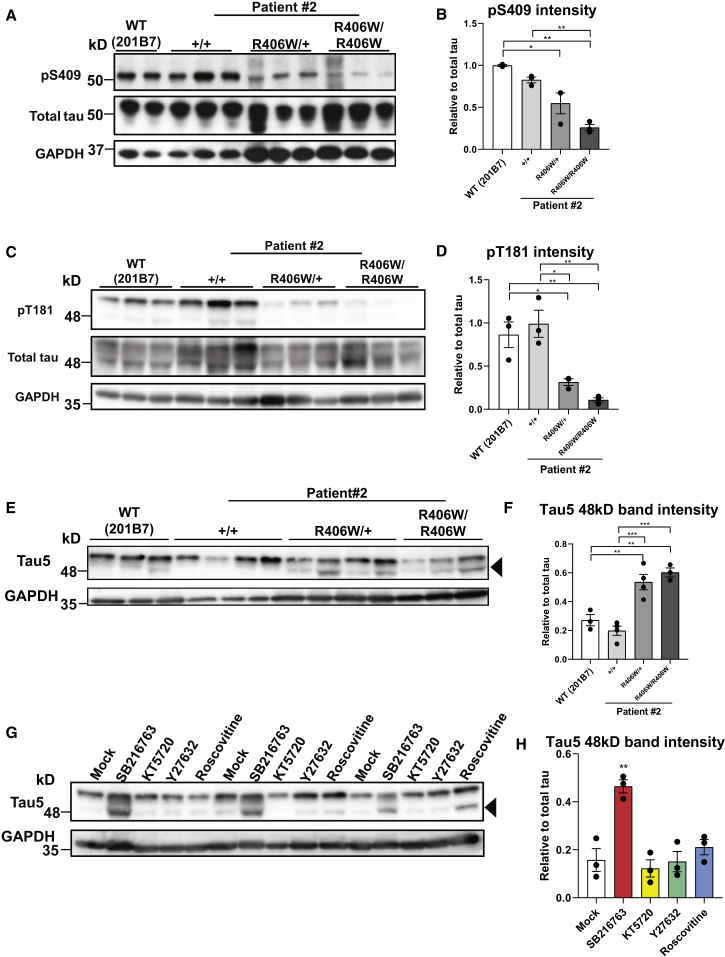
Reduction of Phosphorylation in R406W Mutant Tau by Multiple Kinases (A–D) Western blot analysis investigating tau phosphorylation levels at S409 (A) or T181 (C). Both pS409 and pT181 levels were significantly reduced in the R406W mutant samples (S409, B; T181, D) relative to total tau levels (K9JA) (n = 2–3 independent experiments). (E and F) Western blot analysis with pan-tau antibody Tau5 (E) revealed an increase in the ratio of 48-kDa tau (arrowhead) to total tau (both 48-kDa and 55-kDa bands) in the R406W mutant sample (F) (n = 3–4 independent experiments). (G and H) Western blot analysis investigating the effects of kinase inhibitors SB216763, KT5720, Y27632, and Roscovitine (inhibitors of GSK3β, PKA, RhoK, and CDK5, respectively) on the phosphorylation pattern of tau in WT (201B7) iPSC-derived neurons (G). Quantification of the ratio of 48-kDa tau to total tau (both 48-kDa and 55-kDa bands) revealed an increase of the ratio with SB216763 treatment, a GSK3β inhibitor, in comparison with that with the mock treatment of DMSO (H) (n = 3 independent experiments). Error bars indicate mean ± SEM. One-way ANOVA followed by Tukey's test was performed. ^∗^p < 0.05, ^∗∗^p < 0.01, ^∗∗∗^p < 0.001. See also Figures S2, S3, and S5.

Tau hyperphosphorylation is a key hallmark of tauopathies (Spillantini and Goedert, 2013). Interestingly, western blot analysis revealed that tau phosphorylation at S409 and T181 were lowered in both *MAPT*^R406W/+^ and *MAPT*^R406W/R406W^ neurons compared with the control neurons 30 days after dissociation (Figures 3A–3D, S2A, and S2B). The phosphorylation level of S404 also appeared to decrease when analyzed with an S404 phosphorylation-dependent tau antibody (Figures S2C and S2D), as previously reported (Sakaue et al., 2005). However, reduced phosphorylation was not observed with the PHF1 antibody, which recognizes both S396 and S404 (data not shown), suggesting that the reduction seen with the S404 phosphorylation-specific antibody may simply be reflecting a change in immunogenicity due to the mutation. Tau phosphorylation levels were already reduced even at an earlier time point of 10 days post dissociation (Figures S5A–S5C). Furthermore, co-expression of the glutathione *S*-transferase-conjugated C-terminal fragment of WT or R406W tau with various kinases *in vitro* revealed that the mutation impaired the phosphorylation of S404 by GSK3β and CDK5 and the phosphorylation of S409 by Rho-associated protein kinase (RhoK) and protein kinase A (PKA) (Figure S3A). These findings support the results obtained with the iPSC-derived neurons and suggest that the R406W mutation potentially impairs the phosphorylation of tau by multiple kinases.

In addition, when blotting with pan-tau antibody Tau5, we found that the samples exhibited two major bands (48 kDa and 55 kDa). The intensity of the 48-kDa band was increased in the mutant samples derived from patients #2 and #3 at both 10 days and 30 days after dissociation (Figures 3E, 3F, S2E, S2F, S5D, and S5E). The two bands differed in the phosphorylation level of tau, with the 48-kDa band representing the less phosphorylated form of tau (hereafter termed “hypophosphorylated” tau), since both bands shifted downward and merged into one band representing the 0N3R tau isoform when the samples were treated with λ-phosphatase for dephosphorylation (Figure S2G). Furthermore, the amount of 48-kDa tau increased with GSK3β inhibition using SB216763, which implicates GSK3β as the kinase most responsible for the reduced phosphorylation levels of R406W mutant tau (Figures 3G and 3H). In support of our finding, treatment with CHIR99021, a more specific GSK3β inhibitor, resulted in a dose-dependent increase of the 48-kDa band (Figures S3B and S3C). This is consistent with the results obtained when co-expressing tau and various kinases in COS-7 cells, showing that S404 and S409 are also potential phosphorylation sites of GSK3β and that their phosphorylation by GSK3β is impaired in the R406W mutation (Figures S3D–S3F). Importantly, the phosphorylation level of β-catenin, another major GSK3β substrate, remained unchanged, implying that the reduced tau phosphorylation was not due to a change in GSK3β activity but rather a change in accessibility to the tau protein because of the mutation (Figures S3G and S3H).

The phosphorylation state of tau is known to be regulated by a balance between kinases and phosphatases. To determine whether changes in phosphate activity were involved in the decrease in tau phosphorylation, we measured PP2A activity in our cells, but did not find differences among the control and mutant samples (Figure S3I).

Collectively, our analyses suggest that the R406W mutant tau was less phosphorylated by multiple kinases, with poor phosphorylation by GSK3β particularly accounting for the overall reduction of phosphorylation.

### Increased Fragmentation of R406W Tau by Calpain

**Figure 4:**
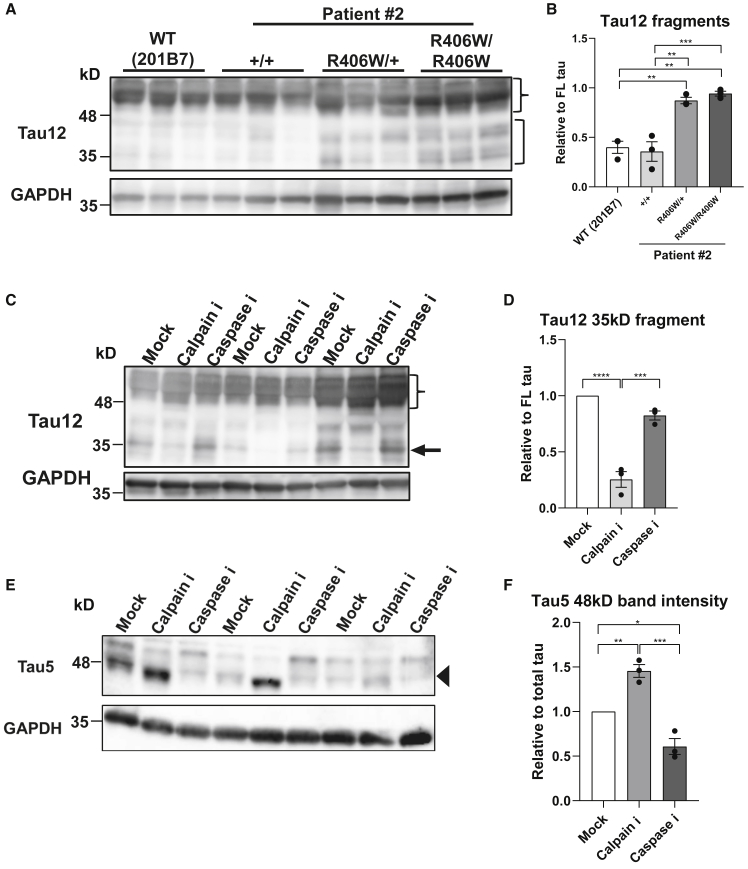
Increased Cleavage of Tau by Calpain Is Dependent on the Phosphorylation State (A and B) Western blot analysis with pan-tau antibody Tau12 (A) revealed increased tau fragments ranging from 35 to 45 kDa (bracket) relative to full-length (FL) tau (brace) in the R406W mutant samples (B) (n = 3 independent experiments). (C and D) Western blot analysis of *MAPT*^R406W/R406W^ samples with Tau12 when treated with 120 μM Z-VAD-FMK (pan-caspase inhibitor), 250 μM ALLN (pan-calpain inhibitor), or DMSO (mock) (C) revealed a decreased ratio of 35-kDa tau fragments (arrow) to full-length (FL) tau (brace) with calpain inhibition (D) (n = 3 independent experiments). (E and F) Western blot analysis of *MAPT*^R406W/R406W^ samples with Tau5 when treated with 120 μM Z-VAD-FMK (pan-caspase inhibitor), 250 μM ALLN (pan-calpain inhibitor), or DMSO (mock) (E) revealed an increased ratio of hypophosphorylated tau (48 kDa; arrowhead) to total tau (both 48-kDa and 55-kDa bands) with calpain inhibition (F) (n = 3 independent experiments). Error bars indicate mean ± SEM. One-way ANOVA followed by Tukey's test was performed. ^∗^p < 0.05, ^∗∗^p < 0.01, ^∗∗∗^p < 0.001, ^∗∗∗∗^p < 0.0001. See also Figure S4.

Tau is reportedly cleaved by several proteases to produce tau fragments of different lengths (Chesser et al., 2013). Western blot analysis revealed an increase in tau fragments ranging from 35 to 45 kDa in the R406W mutant samples across various epitopes (Figures 4A, 4B, and S4A–S4D). This was especially evident when blotting with Tau12, which recognizes the N terminus of tau, implying that the mutation particularly increases the generation of N-terminal tau fragments (Figures 4A and 4B). Next, we attempted to identify the protease generating these fragments. Neurons were treated either with a pan-caspase inhibitor (Z-FAD-VMK) or pan-calpain inhibitor (ALLN). Western blot analysis revealed a decrease of the 35-kDa fragment in samples treated with the calpain inhibitor, but not the caspase inhibitor (Figures 4C and 4D), indicating that calpain was generating the specific fragment. Interestingly, blotting with Tau5 revealed that the amount of hypophosphorylated tau (48 kDa) increased with calpain inhibition (Figures 4E and 4F). We presumed that the hypophosphorylated tau was mainly fragmented by calpain and that the reduction of phosphorylation caused by the R406W mutation made the mutant tau more susceptible to calpain cleavage.

### R406W Mutation Induces Tau Mislocalization and Axonal Dystrophy by Microtubule Destabilization

**Figure 5:**
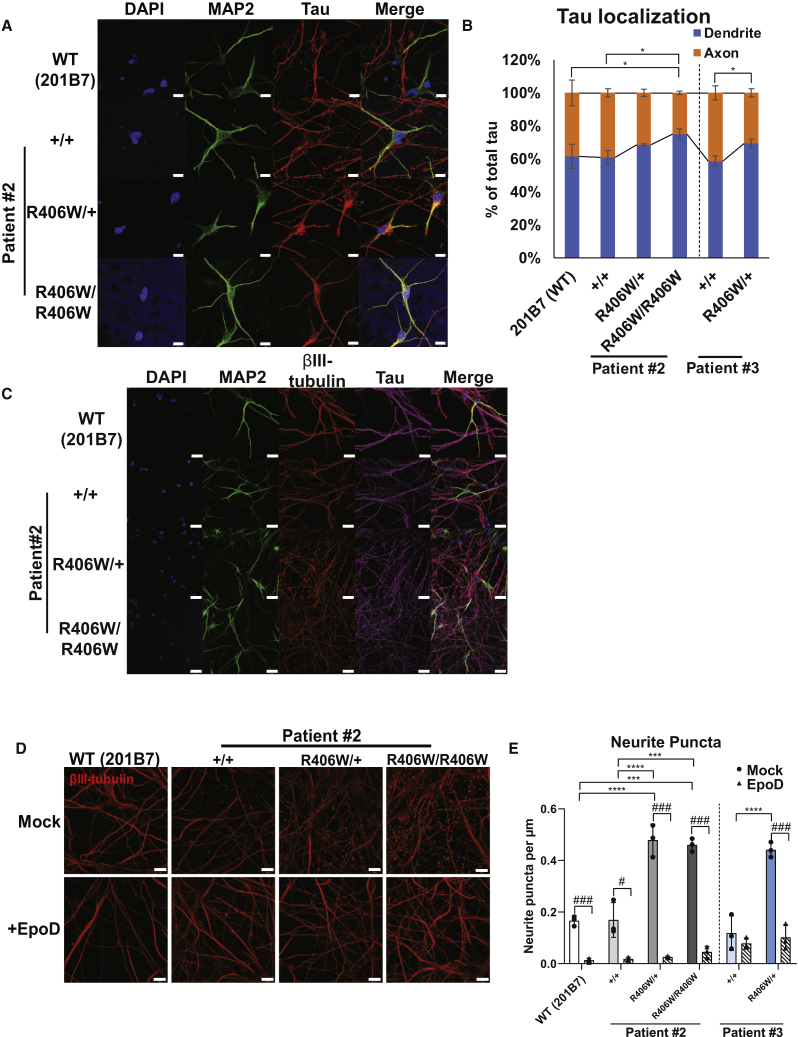
Axonal Phenotypes Caused by MT Destabilization in R406W Mutant Neurons (A and B) Immunostaining of iPSC-derived neurons with MAP2, tau, and DAPI 30 days after dissociation (A). Arrowheads indicate neurons with tau on MAP2^+^ area. Scale bars, 10 μm. The percentage of tau on the dendrites was significantly increased in the mutant neurons when compared with the control neurons (B) (n = 3 independent experiments, ^∗^p < 0.05; one-way ANOVA followed by Tukey's test). (C) Immunostaining of iPSC-derived neurons with MAP2, βIII-tubulin, and tau. Scale bars, 20 μm. (D and E) Representative immunofluorescence pictures of βIII-tubulin^+^ axons with (lower) or without (upper) Epothilone D (EpoD) treatment (D). Scale bars, 10 μm. There was a significantly greater number of puncta in the mutant neurons compared with control neurons, which was rescued with EpoD treatment (E) (n = 3 independent experiments). ^∗∗∗^p < 0.001, ^∗∗∗∗^p < 0.0001 when comparing control with R406W mutants; one-way ANOVA followed by Tukey's test. ^#^p < 0.05, ^###^p < 0.001 when comparing mock with EpoD; Student's t test. Error bars indicate mean ± SEM. See also Figure S5.

Dissociated neurons were further examined for phenotypes at the cellular level. Immunofluorescence of the neurons with tau and MAP2 revealed an increased colocalization of the two markers in the mutant neurons (Figure 5A). Quantitative analysis confirmed that the mutant neurons had a small but significant increase of tau on MAP2-positive dendrites at 30 days after dissociation (Figure 5B). These results suggest that the R406W mutant tau is more likely to mislocalize from the axons to the dendrites of the neurons.

The morphology of the neurons was also investigated with immunofluorescence. Immunostaining with βIII-tubulin revealed a considerable number of dystrophic neurites in the mutant neurons (Figure 5C). Such a staining pattern was not observed with MAP2, indicating that the axons, but not the dendrites, were undergoing degeneration. At 30 days post dissociation, the axons of the mutant neurons consisted of a significantly greater number of small puncta (Figures 5D and 5E). Tau mislocalization and axonal dystrophy were not observed at an earlier time point of 10 days post dissociation (Figures S5F and S5G).

Interestingly, there were fewer axonal puncta when the mutant neurons were treated with Epothilone D (EpoD), a microtubule (MT) stabilizer (Figures 5D and 5E). Altogether, these results suggest that the R406W mutant tau may cause axonal degeneration by altering MT dynamics and/or stability.

### MT Destabilization Disrupts Mitochondrial Transport in the Mutant Neurons

**Figure 6:**
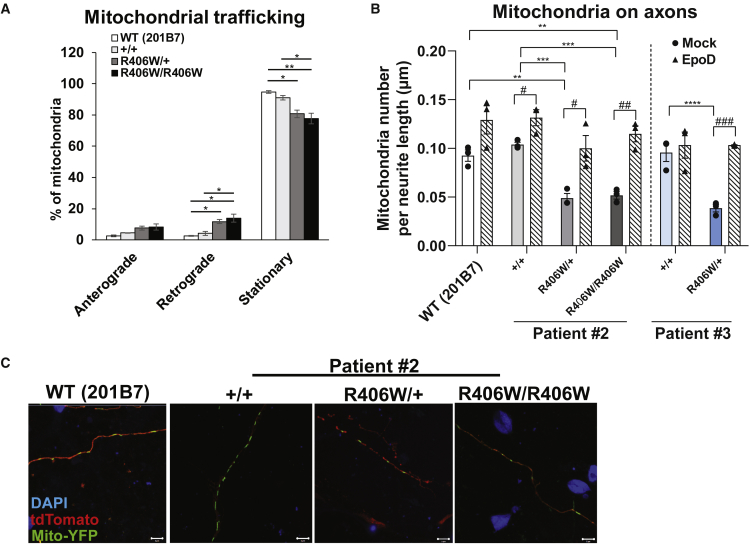
Mitochondrial Transport Defects in R406W Mutant Neurons (A) Quantification of live-imaging mitochondrial trafficking revealed mitochondria in the mutant neurons (patient #2) to be less stationary and moving more in the retrograde direction, when compared with those in the control neurons (n = 3 independent experiments; ^∗^p < 0.05, ^∗∗^p < 0.01, two-way ANOVA followed by Tukey's test). (B and C) Representative pictures of fluorescent signals from iPSC-derived neurons transfected with Mito-eYFP and tdTomato, and immunostained with DAPI (C). Scale bars, 10 μm. There were fewer mitochondria on the axons of R406W mutant neurons compared with control neurons (B) (n = 3 independent experiments; ^∗∗^p < 0.01, ^∗∗∗^p < 0.001, ^∗∗∗∗^p < 0.0001, one-way ANOVA followed by Tukey's test), which recovered to the control level with EpoD treatment (n = 3 independent experiments; ^#^p < 0.05, ^##^p < 0.01, ^###^p < 0.001, Student's t test). Error bars indicate mean ± SEM.

Because axonal degeneration occurred in the mutant neurons, we presumed that the function of the axons in these neurons may be disrupted as well. Axonal transport is one such process responsible for carrying organelles and proteins within neurons. Transport of mitochondria is particularly important, because neurons have high energy demands and require a large supply of ATP to maintain their function and survival (Schwarz, 2013). As such, mitochondrial dysfunction has been implicated during the early stages of multiple neurodegenerative diseases (Lin and Beal, 2006). Thus, we decided to investigate whether there were any defects in mitochondrial transport. After transfecting neurons with Mito-eYFP and tdTomato, we performed live-imaging analysis of the mitochondria in intact axons. We found mitochondria in the mutant neurons to be less stationary and moving more in the retrograde direction (Figure 6A). In support of our finding, fewer mitochondria were in the axons of the mutant neurons compared with those in the WT neurons, which again was rescued with EpoD treatment (Figures 6B and 6C). Overall, these results suggest that the R406W tau-induced MT destabilization caused impairment of the axonal transport machinery.

## Discussion

The role of tau in neurodegeneration has been extensively studied using various models. Development of tau transgenic animal models and cell-line models has provided fundamental insight of tau under both physiological and pathological conditions (Denk and Wade-Martins, 2009, Lim et al., 2014). In the case of R406W mutant tau, hyperphosphorylation and aggregation of tau into filament-like structures (Ikeda et al., 2005, Tatebayashi et al., 2002), as well as retardation of axonal transport (Zhang et al., 2004), has been reported in R406W tau transgenic mice. In cell-line models, soluble R406W tau was less phosphorylated and its binding to microtubules was reduced (Tatebayashi et al., 2006, Matsumura et al., 1999, Dayanandan et al., 1999, Perez et al., 2000, Krishnamurthy and Johnson, 2004). However, the number of studies using a human model has been limited (Imamura et al., 2016, Jiang et al., 2018) and the pathological progression has not been entirely elucidated. Therefore, in this study we developed a patient-derived neuronal model using iPSC technology to recapture the disease pathology induced by the *MAPT* R406W mutation.

Taking advantage of the CRISPR/Cas9 gene-editing technology and the PiggyBac transposase system (Cong et al., 2013, Yusa et al., 2011), we established footprint-free isogenic WT (*MAPT*^+/+^) or homozygous (*MAPT*^R406W/R406W^) iPSC lines from the original heterozygous R406W (*MAPT*^R406W/+^) patient-derived iPSC lines. These lines have a genetically identical background, making them important tools for assessing the genotype-phenotype relationship.

Using previously reported protocols, we generated cerebral organoids from our iPSC lines (Kadoshima et al., 2013, Lancaster et al., 2013). Building on this approach, we developed a method to isolate a homogeneous population of cortical neurons from the organoids. With our protocol, we were able to obtain a high percentage of cells expressing pan-neuronal markers (>85%–90%) and forebrain markers (∼80%) without the use of transgenes (Zhang et al., 2013). There was also a high expression of NeuN, indicating the presence of mature neurons. Furthermore, we can obtain a great number of neurons by dissociating a bulk of organoids, and it is possible to regulate this number by manipulating the number of organoids to be dissociated, making this method feasible for various types of analyses. To our knowledge, this is the first time dissociated organoids were used for disease modeling, and we clearly showed that the dissociated culture is suitable for both biochemical and immunochemical analyses. Hence, we demonstrated the utility of organoids for applications besides 3D modeling.

Using our neuronal culture, we first examined the phosphorylation state of the mutant tau. Abnormal phosphorylation of tau is a pathological hallmark of AD and other tauopathies, which is known to trigger tau pathology by reducing its affinity to microtubules, enhancing aggregation, altering its interactions with other proteins, and missorting it from axons to the somatodendritic compartments in neurons (Wang and Mandelkow, 2016). Several studies have reported the aberrant phosphorylation status of R406W mutant tau, but results have been controversial. Results from *in vitro* experiments and non-neuronal cell lines indicated R406W mutant tau to be less phosphorylated than WT tau (Dayanandan et al., 1999, Matsumura et al., 1999, Perez et al., 2000, Sakaue et al., 2005, Tatebayashi et al., 2006), while studies using murine models or postmortem R406W patient brains claimed that they were hyperphosphorylated (Ikeda et al., 2005, Krishnamurthy and Johnson, 2004, Miyasaka et al., 2001, Tatebayashi et al., 2002). These controversies may have arisen because of the differences in tau solubility and the disease stages reflected in each model. In our human iPSC-derived neuronal model, R406W mutant tau was found to be less phosphorylated at specific epitopes. In addition to demonstrating reduced phosphorylation at T181 and S404, we newly identified S409 to be less phosphorylated in the mutant tau using western blot analyses. Using *in vitro* experiments, we further went on to reveal that the mutation impaired phosphorylation by PKA, RhoK, and GSK3β at these epitopes. In particular, GSK3β accounted for the overall reduction of phosphorylation, which is supported by the fact that T181, S404, and S409 are all potential phosphorylation sites by this kinase (Reynolds et al., 2000). However, it should be noted that the reduction of phosphorylation at S404 is debatable when taking into consideration a change in immunogenicity due to the mutation. Nevertheless, based on our findings and those from previous reports, it is highly plausible that the arginine-to-tryptophan substitution makes tau less prone to phosphorylation by specific kinases, possibly due to some conformational change of the mutant tau. However, as the disease progresses, the mutant tau may become hyperphosphorylated, due to a mechanism not yet identified, as seen in the murine models and patients’ brains (Ikeda et al., 2005, Krishnamurthy and Johnson, 2004, Miyasaka et al., 2001, Tatebayashi et al., 2002).

Results regarding the efficiency of R406W mutant tau to bind and/or assemble microtubules compared with WT tau have also been controversial (DeTure et al., 2000, Hong et al., 1998, Dayanandan et al., 1999, Perez et al., 2000, Hasegawa et al., 1998, Krishnamurthy and Johnson, 2004). Here, we demonstrated that the mutant tau induces cellular phenotypes through the destabilization of microtubules. These phenotypes were rescued by treatment with the microtubule stabilizer EpoD, suggesting that the R406W mutation exerts negative effects on the physiological interaction of tau with microtubules.

Investigation at the cellular level revealed the mislocalization of tau to the dendrites and more punctuate axons in the mutant neurons, both of which have been observed in AD brains (Kowall and Kosik, 1987). Furthermore, there was a decrease in the number of mitochondria on the axons of the mutant neurons. Impairment of mitochondrial distribution has also been reported in an rTg4510 mouse model and in AD patient brains (Kopeikina et al., 2011), as well as in a P301L tau knockin mouse model, which has also interestingly found elevated amounts of hypophosphorylated tau (Rodríguez-Martín et al., 2016). In addition, live-imaging analysis showed that mitochondria in the mutant neurons were less stationary, and an increased percentage moved more in the retrograde direction away from the axons, which is consistent with the reduction of its number in the axons. This is in contrast to a previous study also using FTD patient-derived iPSC neurons, which reported that mitochondria in the mutant neurons were less mobile (Iovino et al., 2015). This discrepancy may be due to the differences in the mutations analyzed (R406W in this study versus P301L and N279K in Iovino et al., 2015). A previous report has demonstrated that microtubule-bound tau inhibits the motility of kinesin and dynein, and that the microtubule binding domain (MTBD) of tau is sufficient for this inhibition (Dixit et al., 2008). Thus, the impaired association of R406W tau with MTs may have promoted dynein to become overly motile, resulting in the increase in retrograde movement of mitochondria. In support of this, the axonal mitochondrial number in the mutant neurons was rescued with EpoD treatment, confirming that MT destabilization induced by the mutant tau caused the transport defect.

Despite the numerous reports on tau pathology, the pathological cascade leading to neurodegeneration is still unknown. For example, the specific role of aberrant tau phosphorylation during pathological progression remains widely unexplored. In this study, we found that reduced tau phosphorylation makes tau more susceptible to calpain cleavage to generate 35-kDa N-terminal fragments. The calpain cleavage site of tau has been reported to be near or inside the MTBD (Chen et al., 2018, Garg et al., 2011). Therefore, the resultant tau fragment either may have lost its ability to stabilize microtubules or have gained a toxic function to destabilize them, ultimately triggering the axonal phenotypes. Furthermore, although the cellular phenotypes could be observed in the mutant neurons at 30 days after dissociation, they could not be detected at an earlier time point. However, tau hypophosphorylation already occurred at 10 days post dissociation. Thus, abnormal tau phosphorylation seen in this study could be an early hallmark of the disease that triggers the consequent pathological events, such as MT destabilization, axonal dystrophy, and axonal transport defects.

Our study reports a previously unidentified molecular mechanism of tau pathology specifically induced by the R406W mutation. Understanding the cascade of pathological events leading to neurodegeneration using iPSC-derived models will help in defining targets for therapeutic development for a wide variety of tauopathies and other neurodegenerative diseases.

## Experimental Procedures

All experimental procedures for iPSCs derived from patients were approved by the Keio University School of Medicine Ethics Committee (approval no. 20080016).

### Cell Culture

Peripheral blood cells from two patients with the *MAPT* R406W mutation were obtained (Ikeuchi et al., 2011), which were maintained in T cell medium, consisting of KBM502 medium (Kohjin Bio, Saitama, Japan) supplemented with Dynabeads Human T-Activator CD3/CD28 (Thermo Fisher Scientific, Waltham, MA, USA).

iPSCs were maintained on irradiated mouse embryonic fibroblasts or SNL 76/7 feeder cells in iPSC medium, consisting of DMEM/F12 medium (Wako, Osaka, Japan) supplemented with 0.08 mM MEM-Non Essential Amino Acid solution (Sigma-Aldrich, St. Louis, MO, USA), 2 mM L-glutamine, 20% (v/v) Knockout Serum Replacement (Thermo Fisher Scientific), 80 U/mL penicillin, 80 μg/μL streptomycin, 0.1 mM 2-mercaptoethanol, and 10 ng/mL basic fibroblast growth factor (PeproTech, Rocky Hill, NJ, USA). Feeder-free iPSCs were maintained on culture dishes coated with 0.25–0.5 μg/μL iMatrix-511 (Laminin-511E8) (Wako) in StemFit AK02N medium (Ajinomoto, Tokyo, Japan). Medium was changed every day for iPSCs on feeder and every other day for feeder-free iPSCs.

### Generation and Dissociation of Cerebral Organoids

Organoids were generated from the iPSCs as previously described with slight modifications (Figure 2A; Lancaster et al., 2013, Kadoshima et al., 2013; described in Supplemental Experimental Procedures). Day-30 organoids were dissociated into single cells using the Nerve Cell Dissociation Medium A (KAC, Kyoto, Japan). 5 × 10^4^ cells, 1 × 10^5^ cells, and 5 × 10^5^ cells were plated onto 60 μg/mL poly-L-ornithine (Sigma-Aldrich) and 10 μg/mL laminin (R&D Systems, Minneapolis, MN, USA)-coated 96-well, 48-well, and 12-well cell-culture plates, respectively (Greiner Bio-One, Kremsmünster, Austria) and cultured for another additional 30 days in Neurobasal medium supplemented with 1% (v/v) B27 supplement with vitamin A, 0.25% (v/v) Glutamax, and 1% (v/v) penicillin/streptomycin.

### Immunofluorescence

The cells were fixed in 4% (w/v) paraformaldehyde for 15–20 min at room temperature, followed by washing in PBS three times for 5 min each. After permeabilization with 0.2% (v/v) Triton X-100 for 15–20 min, the cells were again washed in PBS three times for 5 min each. Samples were incubated in StartingBlock (TBS) Blocking Buffer (Thermo Fisher Scientific) for 1 h at room temperature. Incubation with primary antibodies was performed overnight at 4°C. The following day, the cells were incubated with secondary antibodies for 1 h at room temperature. The cells were observed with a Zeiss LSM700 confocal microscope. Quantification of the immunofluorescence was performed with an In Cell Analyzer 6000 (GE Healthcare, Little Chalfont, UK).

### Western Blot

Cells were dissociated from the culture plates and dissolved in lysis buffer containing 20 mM HEPES, 1 mM MgCl_2_, 100 mM NaCl, 0.5% NP-40, 1 mM dithiothreitol, 0.4 mM Pefabloc (4-(2-aminoethyl)-benzene-sulfonyl fluoride), 10 μg/mL leupeptin, 10 mM NaF, and 10 mM β-glycerophosphate. After homogenization and sonication, the samples were centrifuged at 15,000 × *g* for 15 min at 4°C. For dephosphorylation, samples were treated with 20 U Lambda Protein Phosphatase (New England Biolabs, Ipswich, MA, USA) for 3 h at 30°C. The protein samples were diluted in 4× Laemmli Buffer (BioRad, Hercules, CA, USA) with 10 mM 2-mercaptoethanol and boiled at 95°C for 5 min. The lysates and the Tau Protein Ladder (rPeptide, Athens, GA, USA) were loaded onto Extra PAGE One Precast Gels 7.5%–12.5% (Nacalai Tesque, Kyoto, Japan). SDS-PAGE was performed at 120 V for 105 min. The proteins separated in the gel were transferred onto an Immobilon-P membrane (EMD Millipore). The membranes were blocked with 5% (w/v) skim milk for 30 min and incubated with antibodies diluted in Can Get Signal Immunoreaction Enhancer Solutions (Toyobo, Osaka, Japan). Signals on the membranes were visualized with the ECL Prime detection kit (GE Healthcare) and the images were acquired on an ImageQuant LAS 4000 (GE Healthcare).

For quantification, we normalized the levels of phosphorylated tau and tau fragments to total tau and full-length tau, respectively (see corresponding figure legends for further details). We have not included the quantification of total tau levels in our study because normalization with the highly variable GAPDH levels among different neuronal lines confounded our results.

### Tau Localization Assay

The localization of tau was analyzed with an In Cell Analyzer 6000 (GE Healthcare). Dissociated neurons were immunostained with MAP2, βIII-tubulin, and tau, and the percentage of tau on MAP2- and βIII-tubulin-positive regions was quantified. Dendritic and axonal tau was defined as tau on MAP2-positive area and tau on βIII-tubulin-positive but MAP2-negative area, respectively.

### Neurite Puncta Count

Confocal images of the neurites were obtained with the Zeiss LSM700 oil immersion 100× objective. The number of puncta on neurites was counted manually. For normalization, the length of the neurites was measured using the Fiji plugin Simple Neurite Tracer. Cultures were treated with or without 20 nM EpoD (Abcam) for 24 h for the rescue experiment.

### Mitochondria Count

To visualize mitochondria, we transfected the dissociated neurons with pcDNA3 Mito-eYFP (kindly provided by Drs. T. Tomiyama and T. Umeda, Osaka City University) and tdTomato expression vectors. Confocal images of the mitochondria on the axons were taken with a Zeiss LSM700 oil immersion 63× objective. The numbers of mitochondria were counted manually. For normalization, the length of the neurites was measured using the Fiji plugin Simple Neurite Tracer.

### Live-Imaging Mitochondrial Trafficking

Transfected neurons were visualized with the Olympus FV3000 confocal laser scanning microscope with a silicon-oil immersion 63× objective. A 1.6×–2.0× digital zoom was used for better viewing. Images were taken at 5-s intervals for a total of 100 frames.

### Statistical Analysis

All data from at least three independent experiments are expressed as means ± SEM. All replicates are from different batches of organoids that have been dissociated in separate experiments. GraphPad Prism 8 was used for statistical analysis. The normality of data was assessed using the Shapiro-Wilk test and variances were compared using the Brown-Forsythe test. Data were analyzed with the unpaired, two-tailed Student's t test and one- or two-way ANOVA followed by Tukey-Kramer multiple-comparisons test, as indicated in the figure legends. Differences were considered significant when p < 0.05.

## Author Contributions

M.N., S.S., and H.O. conceived and designed the project. M.N., D.T., M.A., F.K., and S.Y. performed the experiments and analyzed the data. M.N., S.S., and H.O. interpreted the data and wrote the manuscript. D.T., M.A., H.W., S.M., T.K., T.M., N.S., C.M.K., S.H., and T.I. provided cells, reagents, and materials. D.T., M.A., H.W., S.M., T.K., T.M., A.T., N.S., S.H., and K.K. provided technical assistance and critical discussions.

## Supplemental Information

Document S1. Supplemental Experimental Procedures, Figures S1–S5, and Table S1

Document S2. Article plus Supplemental Information
