# Supplementary material for: Pathological Progression Induced by the Frontotemporal Dementia-Associated R406W Tau Mutation in Patient-Derived iPSCs
Source: Stem Cell Reports. 2019 Sep 19;13(4):684–99. doi: 10.1016/j.stemcr.2019.08.011 (PMC6829766; doi:10.1016/j.stemcr.2019.08.011)
Supplement: Document S1. Supplemental Experimental Procedures, Figures S1–S5, and Table S1 [file mmc1.pdf]

Stem Cell Reports, Volume *13*

Supplemental Information

Pathological Progression Induced by the Frontotemporal Dementia-Associated R406W Tau Mutation in Patient-Derived iPSCs

Mari Nakamura, Seiji Shiozawa, Daisuke Tsuboi, Mutsuki Amano, Hirotaka Watanabe, Sumihiro Maeda, Taeko Kimura, Sho Yoshimatsu, Fumihiko Kisa, Celeste M. Karch, Tomohiro Miyasaka, Akihiko Takashima, Naruhiko Sahara, Shin-ichi Hisanaga, Takeshi Ikeuchi, Kozo Kaibuchi, and Hideyuki Okano

Figure S1

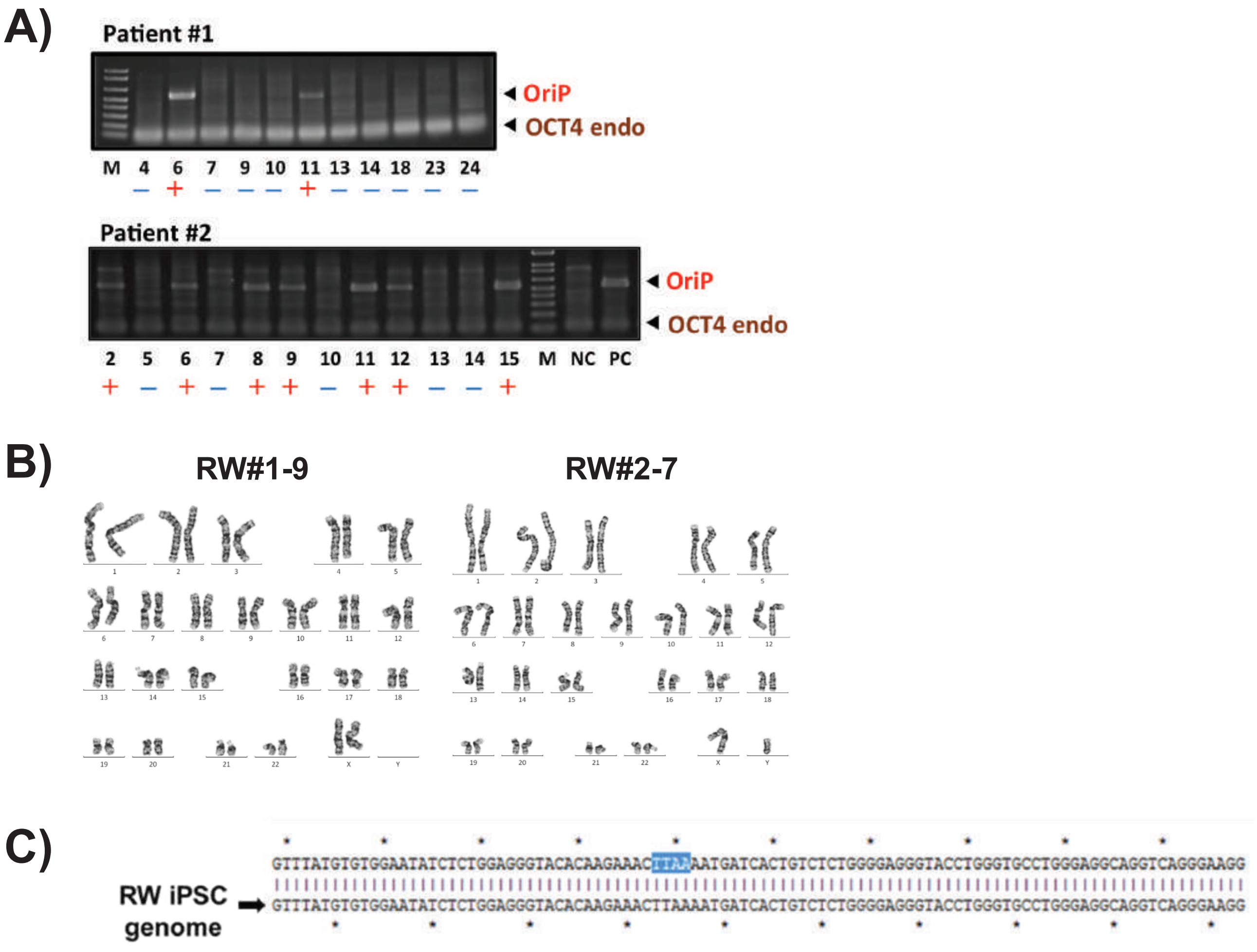
A)
Patient #1
OriP
OCT4 endo
M 4 6 7 9 10 11 13 14 18 23 24
– + – – – + – – – – –
Patient #2
OriP
OCT4 endo
2 5 6 7 8 9 10 11 12 13 14 15 M NC PC
+ – + – + + – + + – – +
B)
RW#1-9
RW#2-7
C)
RW iPSC genome
GTTTATGTGTGGAATATCTCTGGAGGGTACACAAGAAACTTAAAATGATCACTGTCTCTGGGGAGGGTACCTGGGTGCCTGGGAGGCAGGTCAGGGAAGG
GTTTATGTGTGGAATATCTCTGGAGGGTACACAAGAAACTTAAAATGATCACTGTCTCTGGGGAGGGTACCTGGGTGCCTGGGAGGCAGGTCAGGGAAGG

Supplementary Figure 1. Generation of footprint-free iPSC clones with episomal vectors. Related to Figure 1.

(A) PCR analysis evaluating the removal of episomal vectors from the iPSC clones (P=passage number). Arrowheads indicate the bands for OriP (upper) from the episomal vector, and endogenous OCT4 (lower). 9 out of 11 clones and 5 out of 16 clones were free of episomal vectors for patient #1 and #2, respectively.

(B) G-banding karyotype analysis of representative iPSC clones.

(C) DNA sequence of iPSC clone confirming the excision of the selection cassette without any unintended mutations. The data representatively shows an iPSC clone derived from patient #1 (RW#1-9)

Figure S2

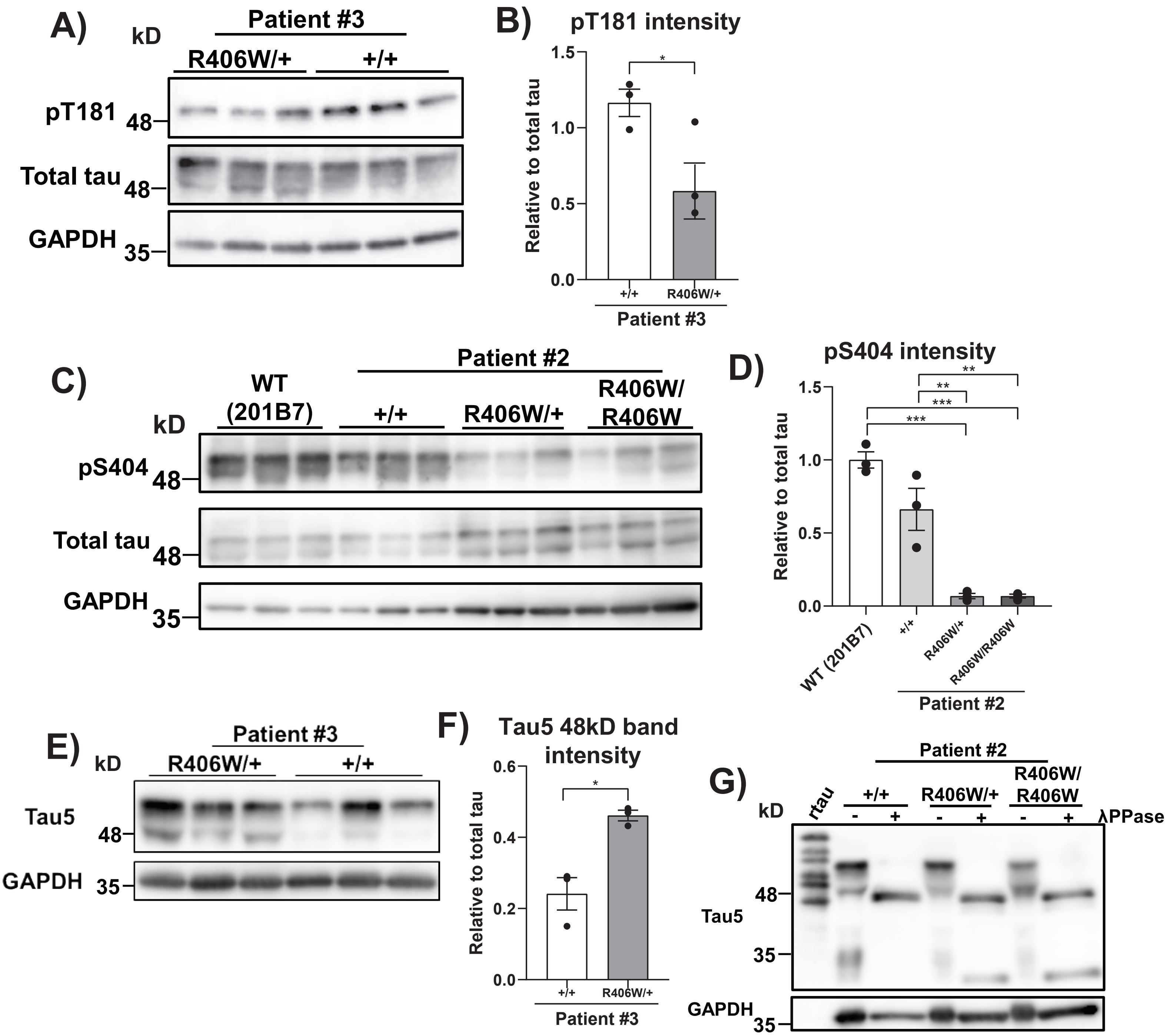
A)
Patient #3
kD
R406W/+
+/+
pT181
48
Total tau
48
GAPDH
35
B)
pT181 intensity
Relative to total tau
1.5
1.0
0.5
0.0
*
+/+
R406W/+
Patient #3
C)
Patient #2
WT (201B7)
+/+
R406W/+
R406W/ R406W
kD
pS404
48
Total tau
48
GAPDH
35
D)
pS404 intensity
Relative to total tau
1.5
1.0
0.5
0.0
**
**
***
***
WT (201B7)
+/+
R406W/+
R406W/R406W
Patient #2
E)
Patient #3
kD
R406W/+
+/+
Tau5
48
GAPDH
35
F)
Tau5 48kD band intensity
Relative to total tau
0.6
0.4
0.2
0.0
*
+/+
R406W/+
Patient #3
G)
Patient #2
rtau
+/+
R406W/+
R406W/ R406W
kD
- + - + - +
λPPase
48
35
Tau5
GAPDH
35

Supplementary Figure 2. Phosphorylation analysis of R406W mutant tau. Related to Figure 3.

(A-B) Western blot analysis investigating tau phosphorylation level at T181 (A). pT181 levels were significantly reduced in the R406W mutant samples derived from patient #3 relative to total tau (K9JA) (B) (n=3 independent experiments; one-way ANOVA followed by Tukey`s test).

(C-D) Western blot analysis investigating tau phosphorylation level at S404 (C). pS404 level was significantly reduced in the R406W mutant samples relative to total tau (K9JA) (D) (n=3 independent experiments; one-way ANOVA followed by Tukey`s test).

(E-F) Western blot analysis of Patient #3 iPSC-derived neurons with pan-tau antibody Tau5 (E) revealed an increase in the ratio of 48kD tau (lower band; arrowhead) to total tau (both 48kD and 55kD bands) (F) (n=3 independent experiments; Student`s t-test).

(G) Representative western blot analysis with total tau antibody Tau5 with or without dephosphorylating the samples with λ-phosphatase. The downwards shift of the 48kD and 55kD bands after dephosphorylation indicates that both bands represent phosphorylated forms of tau with different degrees of phosphorylation. Error bars indicate mean±SEM; *$p<0.05$, **$p<0.01$, ***$p<0.001$.

Figure S3

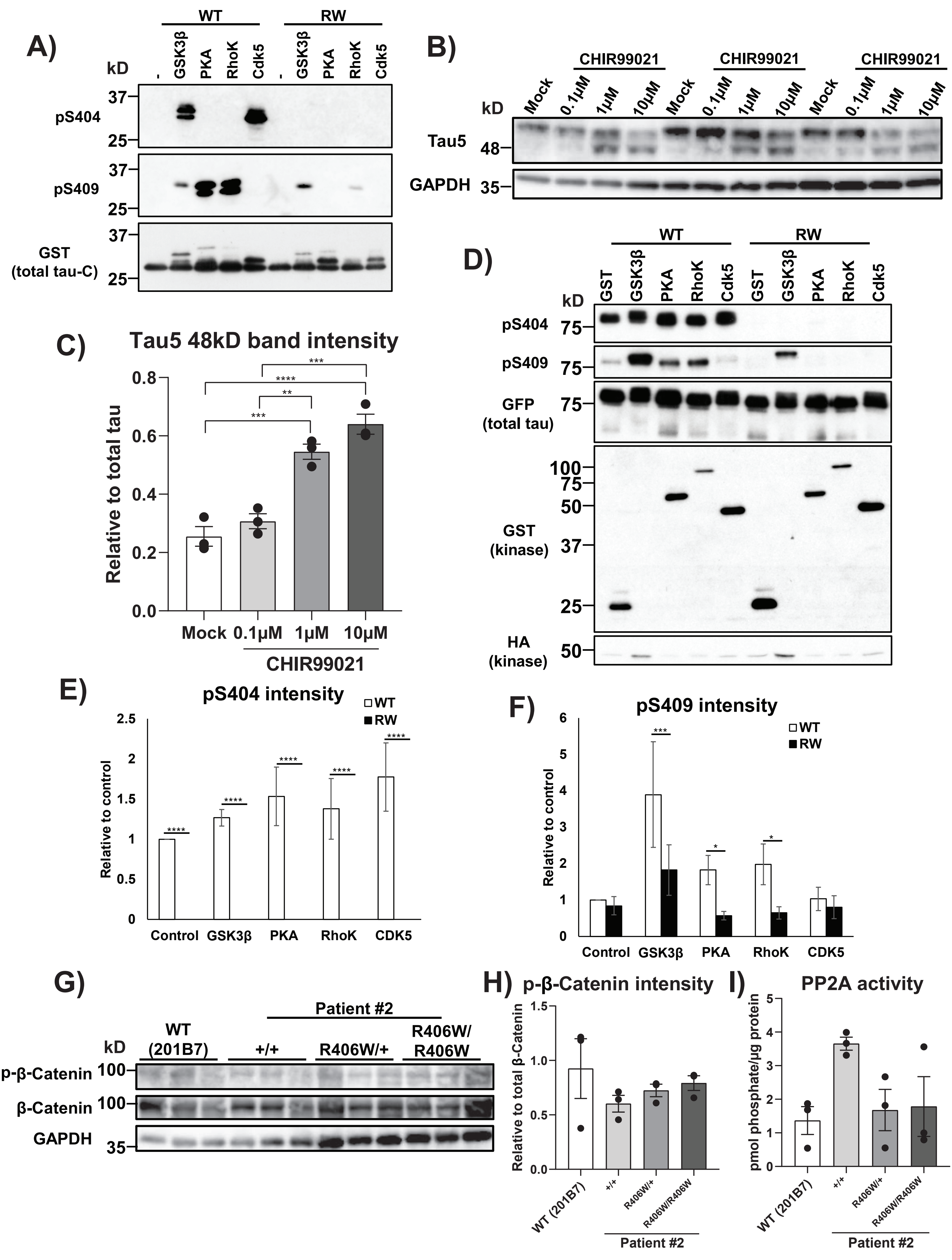

A)
WT
RW
kD
-
GSK3β
PKA
RhoK
Cdk5
pS404
pS409
GST
(total tau-C)
37
25
B)
CHIR99021
Mock
0.1μM
1μM
10μM
Tau5
GAPDH
48
35
C)
Tau5 48kD band intensity
Relative to total tau
0.0
0.2
0.4
0.6
0.8
Mock
0.1μM
1μM
10μM
CHIR99021
D)
GST
pS404
pS409
GFP
(total tau)
GST
(kinase)
HA
(kinase)
100
75
50
37
25
E)
pS404 intensity
WT
RW
Relative to control
Control
GSK3β
PKA
RhoK
CDK5
F)
pS409 intensity
G)
Patient #2
WT
(201B7)
+/+
R406W/+
R406W/
R406W
p-β-Catenin
β-Catenin
GAPDH
H)
p-β-Catenin intensity
Relative to total β-Catenin
WT (201B7)
R406W/R406W
I)
PP2A activity
pmol phosphate/μg protein

Supplementary Figure 3. Effect of kinases on the phosphorylation of R406W mutant tau. Related to Figure 3.

(A) Western blot analysis of the C-terminus fragment of WT and R406W (RW) tau after incubation with each respective kinase. In WT tau, S404 was directly phosphorylated by GSK3β and Cdk5, whereas S409 was directly phosphorylated by PKA and Rho-kinase (RhoK), both of which were impaired by the R406W mutation. GSK3β weakly phosphorylated WT and RW tau at S409 to a similar extent.

(B-C) Western blot analysis investigating the dose-dependent effects of GSK3β inhibitor CHIR99021 on the phosphorylation pattern of tau in WT (201B7) iPSC-derived neurons (B). Quantification of the ratio of 48kD tau to total tau (both 48kD and 55kD bands) revealed a dose-dependent increase of the ratio with CHIR99021 treatment (C) (n=3 independent experiments; one-way ANOVA followed by Tukey`s test).

(D-F) Western blot analysis of WT and R406W (RW) mutant tau conjugated with GFP and co-expressed with the respective kinases in COS-7 cells with tau antibodies detecting phosphorylation at S404 or S409 (D) and quantification of the phosphorylation levels (S404, E; S409, F). All of the kinases enhanced phosphorylation at S404 in WT tau, but no basal phosphorylation at S404 could be detected in RW tau, and none of the kinases were able to enhance the phosphorylation level (E). Co-expression of PKA or Rho-kinase enhanced the phosphorylation of WT tau at S409, but not with RW tau (F) (n=3 independent experiments; Student`s t-test).

(G-H) Western blot analysis of β-Catenin and p-β-Catenin (S33/S37/T41) levels in the iPSC-derived neurons (G). p-β-Catenin levels did not differ significantly among neuronal lines (H) (n=3 independent experiments).

(I) Measurement of phosphatase activity in the iPSC-derived neurons. The amount of phosphate released did not differ among neuronal lines, indicating that there was no significant difference in phosphatase activity (n=3 independent experiments).

Error bars indicate mean±SEM; *$p<0.05$, **$p<0.01$, ***$p<0.001$, ****$p<0.0001$.

Figure S4

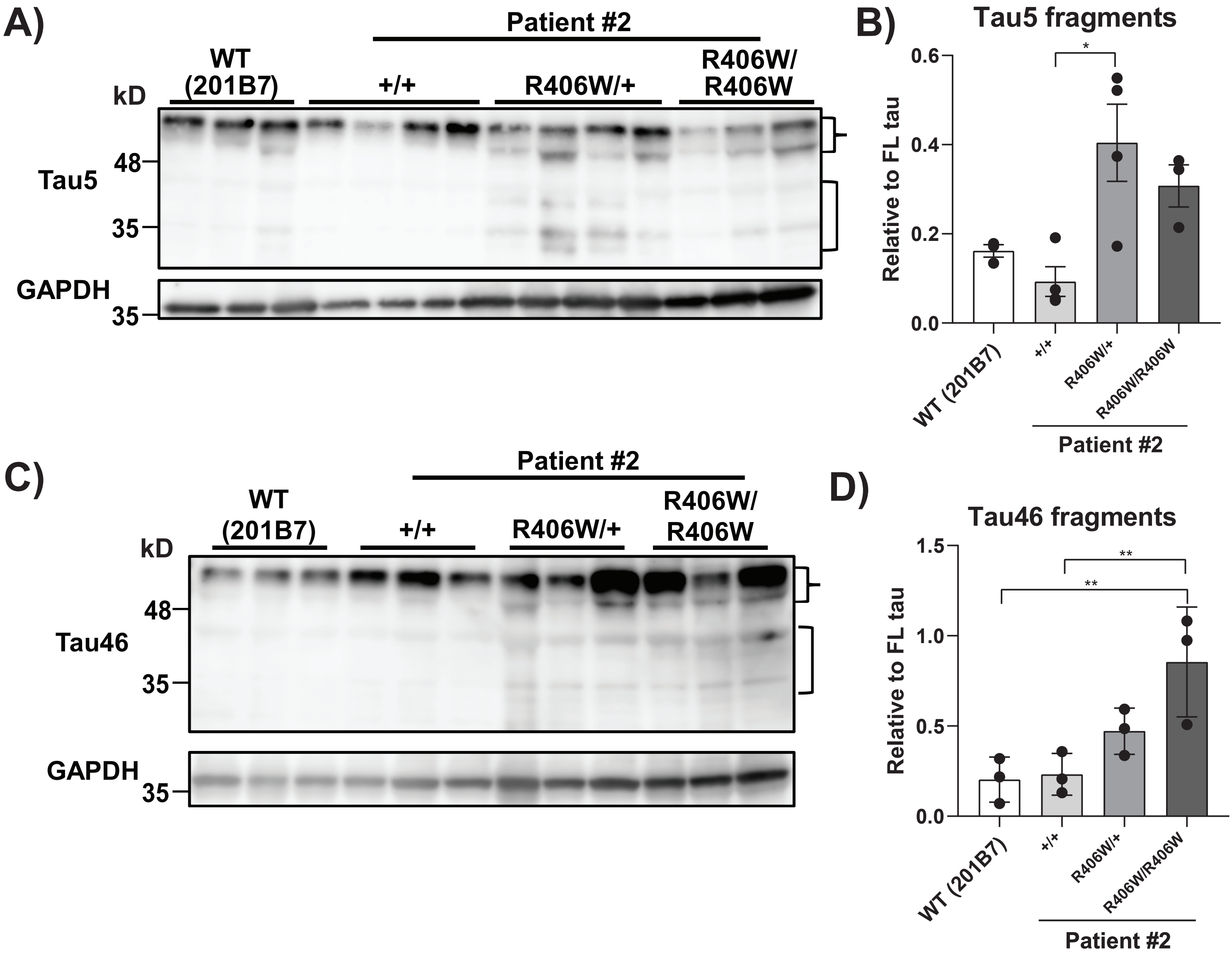
A)
Patient #2
WT (201B7)
+/+
R406W/+
R406W/ R406W
kD
48
Tau5
35
GAPDH
35
B)
Tau5 fragments
Relative to FL tau
0.6
0.4
0.2
0.0
*
WT (201B7)
+/+
R406W/+
R406W/R406W
Patient #2
C)
Patient #2
WT (201B7)
+/+
R406W/+
R406W/ R406W
kD
48
Tau46
35
GAPDH
35
D)
Tau46 fragments
Relative to FL tau
1.5
1.0
0.5
0.0
**
**
WT (201B7)
+/+
R406W/+
R406W/R406W
Patient #2

Supplementary Figure 4. Tau fragmentation analysis in iPSC-derived neurons. Related to Figure 4.

(A-D) Western blot analysis of iPSC-derived neurons with total tau antibodies Tau5 (A) and Tau46 (C) revealed an increase in the ratio of tau fragments (bracket) to full-length tau (brace; tau5, B; tau46, D) ($n=3\sim4$ independent experiments, mean±SEM; *$p<0.05$, **$p<0.01$; One-way ANOVA followed by Tukey`s test).

Figure S5

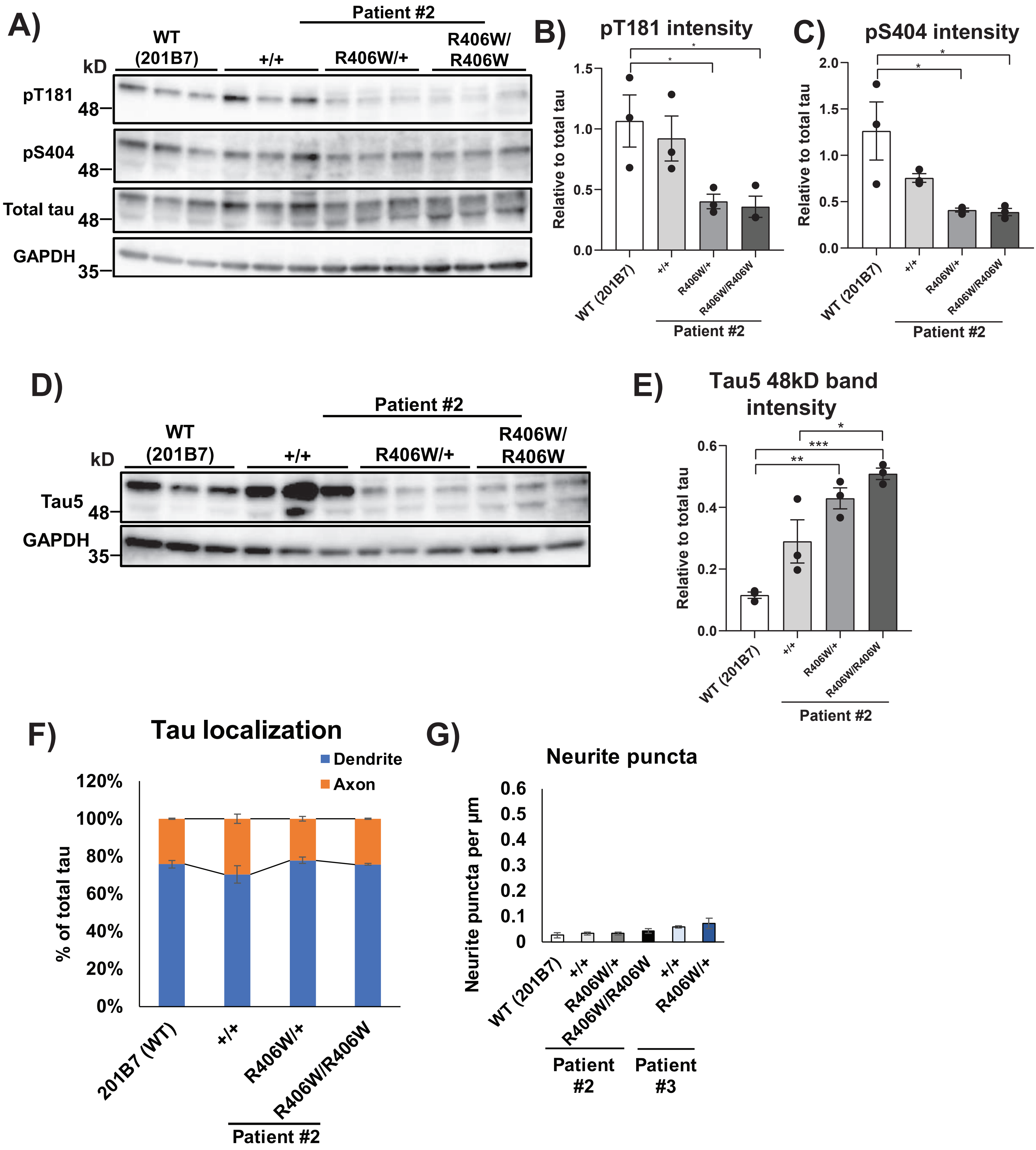

A)
Patient #2
WT (201B7)
+/+
R406W/+
R406W/ R406W
kD
pT181
48
pS404
48
Total tau
48
GAPDH
35
B)
pT181 intensity
Relative to total tau
1.5
1.0
0.5
0.0
*
*
WT (201B7)
+/+
R406W/+
R406W/R406W
Patient #2
C)
pS404 intensity
Relative to total tau
2.0
1.5
1.0
0.5
0.0
*
*
WT (201B7)
+/+
R406W/+
R406W/R406W
Patient #2
D)
Patient #2
WT (201B7)
+/+
R406W/+
R406W/ R406W
kD
Tau5
48
GAPDH
35
E)
Tau5 48kD band intensity
Relative to total tau
0.6
0.4
0.2
0.0
*
***
**
WT (201B7)
+/+
R406W/+
R406W/R406W
Patient #2
F)
Tau localization
Dendrite
Axon
% of total tau
120%
100%
80%
60%
40%
20%
0%
201B7 (WT)
+/+
R406W/+
R406W/R406W
Patient #2
G)
Neurite puncta
Neurite puncta per µm
0.6
0.5
0.4
0.3
0.2
0.1
0
WT (201B7)
+/+
R406W/+
R406W/R406W
+/+
R406W/+
Patient #2
Patient #3

Supplementary Figure 5. Phenotype analysis of iPSC-derived neurons at earlier timepoints. Related to Figure 3, 4, 5.

(A-C) Western blot analysis investigating tau phosphorylation levels at T181 and S404 (A) in iPSC-derived neurons 10 days post dissociation. Phosphorylation levels at both epitopes were significantly reduced in the R406W mutant samples at this timepoint (T181, B; S404, C) relative to total tau (K9JA) (n=3 independent experiments).

(D-E) Western blot analysis with total tau antibody Tau5 (D) revealed an increase in the ratio of 48kD tau (arrowhead) to total tau (both 48kD and 55kD bands) in the R406W mutant samples (E) in iPSC-derived neurons 10 days after dissociation (n=3 independent experiments).

(F) Quantification of the percentage of tau on axons or dendrites of the neurons. The percentage of tau in either of the compartments did not significantly differ among neuronal lines (n=3 independent experiments).

(G) Quantification of the number of axonal puncta per neurite length (μm). The number of axonal puncta did not significantly differ among neuronal lines (n=3 independent experiments).

Error bars indicate mean±SEM. One-way ANOVA followed by Tukey`s test was performed; *$p<0.05$, **$p<0.01$, ***$p<0.001$.

Table S1. iPSC donor information Related to Figure 1.

	Patient #1*	Patient #2*	Patient #3
Gender	Female	Male	Female
Age at onset	50	47	N/A
Age at examination	67	48	N/A
Age at biopsy	N/A	N/A	70
Origin	Peripheral mononuclear blood cell	Peripheral mononuclear blood cell	Fibroblast
Reprogramming method	Episomal	Episomal	Sendai virus
Gene-editing	WT, homo	WT, homo	WT

N/A=not available, WT=wild-type, homo=homozygous

*Modified from Ikeuchi et al., 2011

Supplemental Experimental Procedures

Generation of iPSCs from the patient blood cells

iPSCs were generated from peripheral blood mononuclear cells (PBMCs) or T cells as previously described (Okita et al., 2013, Ichiyanagi et al., 2016, Nakamoto et al., 2018). Briefly, PBMCs were isolated by centrifuging at 15000 g for 20 minutes at room temperature, using the BD VACUTAINER CPT (BD, Franklin Lakes, NJ, USA). Remaining PBMCs were maintained in T cell medium and cultured as T cells. Episomal vectors pCE-hOCT3/4, pCE-hSK, pCE-hUL, pCE-mP53DD (all 0.63 μg), which express OCT3/4, SOX2 and KLF4, L-MYC and LIN28, and p53 dominant negative mutant, respectively, as well as pCXB-EBNA1 (0.5 μg), which express EBNA1 (AddGene IDs: 41813, 41814, 41855, 41856, 41857) were electroporated into 3×10^6 PBMCs or T cells using a Nucleofector 2b Device (Lonza, Basel, Switzerland, V-024 program) with an Amaxa Human T-cell Nucleofector kit (Lonza) according to the manufacturer's instructions. 2 days after transfection, an equal volume of iPSC medium was added to the culture without aspirating the T cell medium. The medium was completely replaced with iPSC medium 4 days after transfection. Colonies with a human embryonic stem cell (ESC)-like morphology were picked up between 26~33 days after transfection and cultured further for cultivation and evaluation. All experimental procedures for iPSCs derived from patients were approved by the Keio University School of Medicine Ethics Committee (approval no. 20080016).

Generation of cerebral organoids

Organoids were generated from the iPSCs as previously described with slight modifications (Figure 2A; Lancaster et al., 2013; Kadoshima et al., 2013). Briefly, on day 0 of organoid culture, iPSCs were dissociated and 3×10^4 cells were aggregated into embryoid bodies (EBs) in low attachment V-shaped 96-well plates (Sumitomo Bakelite, Tokyo, Japan). For 6 days, the EBs were maintained in StemFit AK02N medium supplemented with 30 μM Y-27632, as well as 5 μM SB431542 and 2.5 μM IWP-2 to specifically induce them into the forebrain region of neural tissue. On day 6, the medium was changed to neural induction medium consisting of 1% (v/v) N2 Supplement (Thermo Fisher Scientific), 1% (v/v) Glutamax (Thermo Fisher Scientific), 1% (v/v) MEM-NEAA, 2.5 μM IWP-2, 5 μg/ml sodium heparan sulfate (Sigma-Aldrich), and 1% (v/v) penicillin/streptomycin in DMEM/Ham`s F-12 medium (Thermo Fisher Scientific). On day 9, the EBs were transferred into Ultra-Low Attachment surface 6 well plates (Corning, Corning, NY, USA), grown in differentiation media composed of a 1:1 mixture of DMEM/Ham`s F-12 medium and Neurobasal medium (Thermo Fisher Scientific), supplied with 1% (v/v) N2 supplement, 2% (v/v) B27 supplement without vitamin A (Thermo Fisher Scientific), 100 μM 2-mercaptoethanol, 2.5 μg/ml insulin (Wako), 1% (v/v) Glutamax, 0.5% (v/v) MEM-NEAA, and 1% (v/v) Matrigel (Corning). The plates containing the EBs were maintained on a shaker to enhance nutrient and oxygen absorption. On day 15, the medium was changed to differentiation medium consisting of B27 supplement with vitamin A (Thermo

Fisher Scientific). The medium was changed every 5~7 days from then on.

Pluripotency check of iPSC clones

The iPSC clones were immunostained for pluripotency markers alkaline phosphatase using SIGMAFAST BCIP/NBT (Sigma-Aldrich) according to the manufacturer's instructions, and anti-TRA-1-60 (mouse, 1:500 dilution, EMD Millipore, Billerica, MA, USA).

Karyotype analysis

G-banding analysis of the iPSC lines was conducted by the LSI Medience Corporation (Tokyo, Japan).

Construction of targeting vectors and Cas9 vector

The *MAPT* locus containing exon 13 with or without the R406W mutation was cloned from the patient genome into the pCR-Blunt II-TOPO vector (Thermo Fisher Scientific) by PCR using the following primers: forward TTTTTGGCTTTACCAGATGCTCA, reverse TCATTACTGAGAAGGGGTGGTGA. The PCR conditions are as follows: 35 cycles of 98℃ for 10s, followed by 68℃ for 3 min 30s. The PrimeSTAR Max DNA Polymerase (Takara, Kusatsu, Japan) was used. A 2-kb 5′arm and 5-kb 3′arm consisting of exon 13 with the mutation site were cloned individually into separate entry vectors by PCR using the following primers: 5′arm forward, AACTTTGTATAATAAAGTTGTGACTGAGTGGGTCTGGATAGG, 5′arm reverse, CAGACTATCTTTCTAGGGTTAAGTTTCTTGTGTACCCTCCAG, 3′arm forward, ATGATTATCTTTCTAGGGTTAAAATGATCACTGTCTCTGGGG, 3′arm reverse, CAACTTTGTATAGAAAAGTTGCTAAGGGTGCGTGGGAAAGA. The PCR conditions are as follows: 98℃ for 1 min, followed by 20 cycles of 98℃ for 10s and 68℃ for 2 min (5′arm) or 5 min (3′arm). Using the GeneArt Seamless Cloning and Assembly Kit (Thermo Fisher Scientific), these entry vectors, along with another entry vector consisting of a selection cassette with Puromycin-ΔTK driven by a mouse PGK promoter and PiggyBac inverse terminal repeats (ITRs) at both ends (Figure 2A), were assembled into a single destination vector.

The Cas9 expression plasmid vector pSPCas9(BB)-2A-Puro (PX459) was obtained from AddGene (ID: 48139). Complementary oligonucleotides encoding sgRNA were annealed and cloned into the *Bbs*I site in the Cas9 vector. The sequences of the oligonucleotides are as follows: upper, CTTAAAATGATCACTGTCTCTGG, lower, AAACGAGACAGTGATCATTTTAAGC.

Transfection of targeting vector into iPSCs

Feeder-free iPSCs were pre-treated with 10 μM Y-27632 (EMD Millipore) overnight to prevent apoptosis induced by dissociation (Watanabe et al., 2007). The iPSCs were dissociated into single cells with Tryple Select (Thermo Fisher Scientific). The targeting vector with or without the R406W mutation and the Cas9

expression vector were electroporated into 1 × 10^6 iPSCs using NEPA21 (NepaGene, Chiba, Japan). Transfected cells were plated onto iMatrix-511-coated 60 mm dishes (Iwaki, Chiba, Japan). Y-27632 was removed from the culture 2 days after the transfection. Cells were treated with 0.75~0.8 μg/μl puromycin on days 2, 3, 6, and 8 after plating.

Colonies with a human ESC-like morphology were picked up and DNA from each colony was extracted using the DNeasy Blood & Tissue Kit (QIAGEN, Hilden, Germany). PCR analysis evaluating the knock-in of targeting vectors was performed using the following primers: forward, GCGTCCCAGAAAGGGTATAGG, reverse GTCACGTAAAAGAATTGTTTGTTGA. The DNA of the clones in which knock-in occurred was analyzed by Sanger sequencing with the 3130xl Genetic Analyzer (Applied Biosystems, Waltham, MA, USA) to check the mutation site.

Excision of PiggyBac cassette from iPSCs

Feeder-free iPSCs pre-treated with 10 μM Y-27632 overnight were dissociated into single cells with Tryple Select (Thermo Fisher Scientifc). 1 μg PBx (Excision Only PiggyBac Transposase; Funakoshi, Tokyo, Japan) was transfected into 1 × 10^6 iPSCs with the GeneJuice Transfection Reagent (EMD Millipore) and plated onto iMatrix-511-coated 10 cm dishes (Iwaki). DNA extraction and PCR analysis were performed as described above.

Generation of cerebral organoids

Organoids were generated from the iPSCs as previously described with slight modifications (Figure 2A; Lancaster et al., 2013; Kadoshima et al., 2013). Briefly, on day 0 of organoid culture, iPSCs were dissociated and 3 × 10^5 cells were aggregated into embryoid bodies (EBs) in low attachment V-shaped 96-well plates (Sumitomo Bakelite, Tokyo, Japan). For 6 days, the EBs were maintained in StemFit AK02N medium supplemented with 30 μM Y-27632, as well as 5 μM SB431542 and 2.5 μM IWP-2 to specifically induce them into the forebrain region of neural tissue. On day 6, the medium was changed to neural induction medium consisting of 1% (v/v) N2 Supplement (Thermo Fisher Scientific), 1% (v/v) Glutamax (Thermo Fisher Scientific), 1% (v/v) MEM-NEAA, 2.5 μM IWP-2, 5 μg/ml sodium heparan sulfate (Sigma-Aldrich), and 1% (v/v) penicillin/streptomycin in DMEM/Ham`s F-12 medium (Thermo Fisher Scientific). On day 9, the EBs were transferred into Ultra-Low Attachment surface 6 well plates (Corning, Corning, NY, USA), grown in differentiation media composed of a 1:1 mixture of DMEM/Ham`s F-12 medium and Neurobasal medium (Thermo Fisher Scientific), supplied with 1% (v/v) N2 supplement, 2% (v/v) B27 supplement without vitamin A (Thermo Fisher Scientific), 100 μM 2-mercaptoethanol, 2.5 μg/ml insulin (Wako), 1% (v/v) Glutamax, 0.5% (v/v) MEM-NEAA, and 1% (v/v) Matrigel (Corning). The plates containing the EBs were maintained on a shaker to enhance nutrient and oxygen absorption. On day 15, the medium was changed to differentiation medium consisting of B27 supplement with vitamin A (Thermo Fisher Scientific). The medium was changed every 5~7 days from then on.

Phosphorylation analysis of tau

Human Tau cDNAs (0N3R, WT and R406W) were synthesized, and subcloned into pEGFP-c1 mammalian expression vector. The C-terminus of Tau (308-351 aa; VVSGDTSP(R/W)HLSNVSSTGSIDMVDSPQLATLADEVSASLAKQGL) was subcloned into pGEX-2T E. coli expression vector. GST-PKACa (#01-127) and GST-GSK3β (#04-141) were purchased from Carna Biosciences (Kobe, Japan). GST-Rho-kinase-cat, GST-CDK5, and GST-p35 were expressed in Sf9 cells using a baculovirus system and purified using glutathione Sepharose beads (Amano et al., 1999).

For *in vitro* analysis, GST-Tau-C (1 μM) was incubated with each kinase (30 nM) in a reaction mixture (25 mM Tris-HCl at pH 7.5, 1 mM EDTA, 1 mM DTT, 5 mM $MgCl_2$, and 50 μM ATP) for 30 min at 30°C. The reaction mixtures were boiled in SDS sample buffer and subjected to western blot analysis.

Plasmids encoding Tau and each kinase were transfected into COS7 cells using the Lipofectamine® 2000 reagent (Thermo Fisher Scientific) according to the manufacturer's protocol. The cells were lysed with SDS sample buffer and subjected to western blot analyses.

Immunofluorescence antibodies

The following primary antibodies and dilutions were used: anti-TBR1 (rabbit, Abcam, Cambridge, UK, ab31940, 1:200 dilution), anti-MAP2 (chicken, Abcam ab5392, 1:10000 dilution), anti-βIII-tubulin (rabbit, Abcam, ab18207, 1:1000 dilution), anti-FOXG1 (rabbit, Abcam, ab18259, 1:500), and anti-tau (RTM38, Fujifilm Wako Pure Chemical, Osaka, Japan; rat, 1:5000 dilution). The following day, the cells were incubated with secondary antibodies for 1 hour at room temperature. Secondary antibodies used include Goat Alexa 488, 555, and 647 conjugates (Thermo Fisher Scientific, 1:500 dilution). Nuclear staining was performed with the Cellstain DAPI solution (DOJINDO, Kumamoto, Japan, 1:1000 dilution) for 15~20 minutes at room temperature.

Western blot antibodies and reagents

The following primary antibodies were used: anti-tau (K9JA) (Agilent Technologies, Santa Clara, CA, USA, 1:10000 dilution), Tau5 (mouse, 1:2000 dilution), Tau12 (mouse, 1:10000 dilution), Tau46 (mouse, Cell Signaling Technology, Beverly, MA, USA, 4019, 1:2000 dilution), anti-Tau-pT181 (rat, Cell Engineering Corporation, Osaka, Japan, 1:1000 dilution), anti-Tau-pS409 (rabbit, 1:1000 dilution; Amano et al., 2003), anti-β-Catenin (rabbit, Cell Signaling Technology, 8480, 1:2000 dilution), anti-p-β-Catenin (S33/S37/T41) (rabbit, Cell Signaling Technology, 9561, 1:1000 dilution), and anti-GAPDH (rabbit, Sigma-Aldrich, G9545, 1:10000 dilution).

For protease inhibition, dissociated neurons were pre-treated with either 120 μM Z-VAD-FMK (Peptide Institute, Osaka, Japan), pan-caspase inhibitor, or 250 μM ALLN (Nacalai Tesque), pan-calpain inhibitor, for 24 hours before cell lysis.

For kinase inhibition, dissociated neurons were pretreated with 1 μM KT5720 (Tocris), 10 μM Y27632 (Merck), 20 μM SB216763 (Tocris), 20 μM Roscovitine (Sigma), or CHIR99021 (0.1 μM, 1 μM, or 10 μM) for 24 hours before cell lysis.

Phosphatase activity assay

Phosphatase activity was measured using the Serine/Threonine Phosphatase Assay System (Promega, Madison, WI, USA) according to the manufacturer's instructions. Briefly, cells were lysed in phosphatase storage buffer, which consisted of 0.05M Tris-HCl (pH7.0), 0.1mM EGTA, 0.1% 2-ME, 0.4 mM Pefabloc (AEBSF), and 10 μg/ml leupeptin and endogenous phosphate was removed as using the spin columns provided in the assay kit. To measure PP2A activity, the reaction premix, which consists of 5x PP2A reaction buffer (250 mM imidazole (pH7.2), 1mM EGTA, 0.1% 2-ME, 0.5mg/ml BSA, 0.4 mM Pefabloc (AEBSF), and 10 μg/ml leupeptin), synthetic phosphopeptide, and the lysate samples, was prepared in duplicate in 96 well plates and allowed to react for 10 min at 37°C. Reaction was stopped with the addition of Molybdate Dye/Additive mixture and the dye color was allowed to develop for 15~30 min at room temperature. Absorbance was measured at 600 nm with the Glomax Microplate Reader (Promega) to assess the amount of phosphate released.

List of plasmids used in this study

Plasmid name	Features
pSPCas9(BB)-2A-Puro (PX459)	Mammalian expression vector, CBh promoter, Cas9
pGEX-2T-Tau-C-WT/R406W	E.coli expression vector, tac promoter, GST-Tau-C (human 0N3R, 308-351 aa)
pEGFP-c1-Tau-WT/R406W	Mammalian expression vector, CMV promoter, EGFP-Tau (human 0N3R, 1-351 aa)
pCGN-HA-GSK3B-CA	Mammalian expression vector, CMV promoter, HA-GSK3B-CA (human)
pEF-BOS-GST-PKA-CA	Mammalian expression vector, EF-1α promoter, GST-PKA-CA (mouse)
pEF-BOS-GST-Rho-kinase-cat	Mammalian expression vector, EF-1α promoter, GST-Rho-kinase catalytic region (bovine)
pEF-BOS-GST-CDK5	Mammalian expression vector, EF-1α promoter, GST-CDK5 (human)
pEF-BOS-GST-p35	Mammalian expression vector, EF-1α promoter, GST-p35 (human)
pcDNA3-Mito-eYFP	Mammalian expression vector, CMV promoter, Mito-eYFP
ptdTomato	Mammalian expression vector, CMV promoter, tdTomato

Supplemental References

Amano, M., Kaneko, T., Maeda, A., Nakayama, M., Ito, M., Yamauchi, T., Goto, H., Fukata, Y., Oshiro, N., Shinohara, A., et al. (2003). Identification of Tau and MAP2 as novel substrates of Rho-kinase and myosin phosphatase. J Neurochem. *87*, 780-790.

Amano, M., Chihara, K., Nakamura, N., Kaneko, T., Matsuura, Y., and Kaibuchi, K. (1999). The COOH terminus of Rho-kinase negatively regulates rho-kinase activity. J. Biol. Chem. *274*, 32418-32424.

Watanabe, K., Ueno, M., Kamiya, D., Nishiyama, A., Matsumura, M., Wataya, T. and Sasai, Y. (2007). A ROCK inhibitor permits survival of dissociated human embryonic stem cells. Nature Biotechnol. *25*, 681-686.
